# A Gold Nanoparticle-Based Cortisol Aptasensor for Non-Invasive Detection of Fish Stress

**DOI:** 10.3390/biom14070818

**Published:** 2024-07-09

**Authors:** Yuki Tanaka, Nur Asinah binte Mohamed Salleh, Marie Ruoyun Tan, Shubha Vij, Caroline Lei Wee, Laura Sutarlie, Xiaodi Su

**Affiliations:** 1 Institute of Materials Research and Engineering, Agency for Science, Technology and Research (A*STAR), 2 Fusionopolis Way, Innovis, #08-03, Singapore 138634, Singapore; tanaka_yuki@imre.a-star.edu.sg (Y.T.); asinah@imre.a-star.edu.sg (N.A.b.M.S.); 2 Republic Polytechnic, School of Applied Science, 9 Woodlands Ave 9, Singapore 738964, Singapore; marie_tan@rp.edu.sg (M.R.T.); shubha_vij@rp.edu.sg (S.V.); 3 Tropical Futures Institute, James Cook University Singapore, 149 Sims Drive, Singapore 387380, Singapore; 4 Institute of Molecular and Cell Biology (IMCB), Agency for Science, Technology and Research (A*STAR), 61 Biopolis Drive, Proteos, Singapore 138673, Singapore; weel@imcb.a-star.edu.sg; 5 Department of Chemistry, National University of Singapore, Block S8, Level 3, 3 Science Drive 3, Singapore 117543, Singapore

**Keywords:** cortisol, aptamer, gold nanoparticle, aptasensor, fish stress

## Abstract

Cortisol is a key stress biomarker in humans and animals, including fishes. In aquafarming, stress monitoring using cortisol quantification can help to optimize aquaculture practices for welfare and productivity enhancement. However, most current methods for cortisol detection rely on invasive tissue sampling. In this work, we developed a gold nanoparticle (AuNP)-based cortisol sensor to address the demand of detecting picomolar ranges of cortisol from complex fish tank water matrices as a non-invasive alternative for more effective stress monitoring. We first identified a DNA aptamer with effective binding to cortisol and then conjugated the thiol-labelled aptamer to AuNPs together with a blocker molecule (CALNN) to form an Au-Apt-CALNN conjugate that is stable in fish tank water. The cortisol detection principle is based on magnesium chloride (MgCl_2_)-induced particle aggregation, where the cortisol-bound aptamer on the AuNPs folds into a tertiary structure and provides greater protection for Au-Apt-CALNN against MgCl_2_-induced aggregation due to steric stabilization. At an optimum MgCl_2_ concentration, the differential stability of particles with and without cortisol binding offers a limit of detection (LOD) of 100 pM for cortisol within a 35 min reaction. The aptasensor has been validated on recirculating aquaculture system (RAS) fish tank water samples by the HPLC method and was able to detect changes in water cortisol induced by two different stress paradigms. This on-site deployable and non-invasive sensor offers opportunities for more efficient and real-time fish stress monitoring for the optimization of aquaculture practices.

## 1. Introduction

In recent years, the detection and monitoring of physiological stress levels have received widespread research interest [1,2,3,4]. Amongst various stress hormones, cortisol is recognized as a vital and reliable clinical biomarker for stress diagnosis in humans and animals, including fishes [5,6,7,8,9,10,11]. In particular, the monitoring of stress levels in farmed fishes is essential for optimizing aquaculture practices for the enhancement of welfare and productivity [12]. To tackle the problem of invasive blood sampling, non-invasive cortisol sensors have been developed mostly for human monitoring, including wearable sensors for sweat cortisol, etc. [2,8,13,14]. In aquafarming, detecting cortisol released into fish tank water is a promising approach for non-invasive stress quantification [8,9]. A good number of studies have been published to confirm the association of water cortisol (in picomolar ranges) with fish stress. However, the cortisol measurements in these studies relied mostly on time consuming conventional methods, e.g., liquid chromatography/ HPLC [7,8,9,15,16] and immunoassays such as ELISA [17,18].

Cortisol biosensors have been mostly developed for human stress monitoring with nanomolar-level sensitivity. They rely on either antibody or DNA aptamers for selective target detection in sandwiched or competitive assays, exploiting various optical and electrochemical signaling methods [19,20,21,22,23]. In comparison to antibodies, DNA aptamers exhibit the advantages of higher stability, lower production cost, and ease of chemical modifications [24,25,26]. Meanwhile, metal nanoparticles, such as gold nanoparticles (AuNPs), possess unique optical properties related to localized surface plasmon resonance (LSPR) that allow AuNPs to be used as signal transducers in designing biosensors [27,28]. The combination of AuNPs and anti-cortisol DNA aptamers has been exploited for cortisol detection through particle aggregation-based colorimetric assays [22,29,30,31], surface plasmonic resonance (SPR) spectra [32,33] and electrochemical means [34,35,36].

By far the largest number of anti-cortisol DNA aptamers have been selected via the Systematic Evolution of Ligands by EXponential enrichment (SELEX) process [37,38,39,40]. Martin et al. first reported an 85 mer aptamer (Apt 15-1, *K*_d_ of 6.9 ± 2.8 μM by equilibrium dialysis and 16.1 ± 0.6 μM by microscale thermophoresis) and its truncated 40 mer counterpart (Apt 15-1a) by removal of two primer-binding regions (*K*_d_ not reported) [37]. In this study, the 85 mer Apt 15-1 was used to detect cortisol by exploiting its protection of AuNPs against salt-induced aggregation and the retarded protection when cortisol was present. The authors reported a selective quantification of cortisol in the range of 150–600 nM, relative to other stress biomarkers (norepinephrine and epinephrine) and a structurally analogous biomarker of liver dysfunction, cholic acid. In another study, Dalirirad et al. used the shorter Apt 15-1a with AuNPs to detect cortisol in sweat, reporting an LOD of 1 ng/mL by exploiting the same physical adsorption of the aptamer onto bare AuNPs for protection from salt-induced aggregation and reduced adsorption when cortisol-bound [31]. The sensor exhibited cortisol selectivity against other hormones, such as neuropeptide Y and serotonin. Furthermore, Sabri et al. performed in silico molecular docking and molecular dynamics simulation to study the interactions between cortisol and ten selected cortisol-binding aptamers [37,41], including Apt 15-1a and other truncated portions of the 85 mer aptamer [39]. Amongst the ten aptamers, Apt 15-1a demonstrated the lowest binding energy (−42.3 kJ/mol), i.e., higher stability, when forming a complex with cortisol [39]. Nonetheless, the Apt 15-1a–cortisol complex has a highly fluctuating average total energy compared to that of the typical stable complex between glucocorticoid receptors and cortisol [39], as indicated by the relatively low complex stability of the Apt 15-1a–cortisol complex. More recently, a few high affinity cortisol-binding aptamers (nM *K*_d_) have been derived through theoretical simulations [38,40]. One example is a 42 mer aptamer (Apt CSS.1) of *K*_d_ = 245 nM, which was further employed alongside a DNA staining dye to detect cortisol with an LOD of 742 nM, with deoxycholic acid, 17β-estradiol, thymidine and dopamine as negative controls [38]. In the same study, the authors reported the absence of cortisol binding (tested up to 10 µM) to the 40 mer Apt 15-1a. The lack of cortisol-binding capability of this 40 mer Apt 15-1a reported in this study is opposed to the earlier reports of it being used for cortisol detection; but to a certain extent resonated with its μM *K*_d_ (very low affinity reported earlier [31,37,39,41]. More recently, the high binding affinity and selectivity of the 42 mer Apt CSS.1 towards cortisol have been further confirmed when compared to a few mutant Apt CSS.1 [42].

While a few AuNP aggregation-based cortisol sensors have been developed using anti-cortisol DNA aptamers, many of them merely exploit bare AuNPs and the physical absorption of the DNA aptamers [24,29,30]. Such principles are not suitable for cortisol detection in fish tank water samples, which contain many compounds such as amino acids [43], inorganic ions [44] and proteins [45] that may interfere with the aggregation of bare AuNPs. Alternatively, the assembly of aptamers (and blocker molecules) through covalent bonds onto nanoparticles can introduce stabilization forces through surface charge and steric protection [46,47]. Wu et al. functionalized AuNPs with thiol-modified 85 mer Apt 15-1 and found that the as-prepared Apt-AuNP conjugates can withstand salt-induced aggregation; however, the stability was reduced upon cortisol binding in a range of 1–1000 nM [30]. They attributed the destabilization effect to the change in aptamer configuration after aptamer-cortisol complex formation but did not provide detailed characterization. In another study of aptamer-AuNPs conjugate binding to molecules of similar molecular weight range (e.g., adenosine), Zhao et al. conjugated thiol-functionalized adenosine aptamers (29 mer) onto AuNP and reported analyte binding-enhanced AuNP stability [46], which is different from the destabilization effect observed in the 85 mer Apt 15-1-AuNPs study upon cortisol binding.

In order to verify the various cortisol-binding outcomes reported for the Apt 15-1a and Apt CSS.1 in this study [31,37,38], we first studied the binding efficiency of Apt 15-1a and Apt CSS.1 to cortisol, exploiting bare AuNPs as colorimetric sensing materials, based on aptamer protection of AuNPs from salt-induced aggregation and the retarded protection by cortisol. Next, differently to most of the early reports, we conjugated thiol-labelled Apt CSS.1 (confirmed for its higher affinity) and a peptide blocker (CALNN) to AuNPs to obtain highly stable AuNP conjugates in different salt mediums, including recirculating aquaculture system (RAS) tank water and artificial seawater (ASW). After that, a “mix-and-measure” assay protocol was developed for cortisol detection via differential aggregation profiles between the aptamer and CALNN co-conjugated AuNPs before and after cortisol binding under a properly selected salt condition. Dynamic light-scattering (DLS) measurement was performed to reveal the mechanism for the analyte binding-induced stabilization principle. With all these optimizations, an LOD of 100 pM was achieved, suitable for fish stress monitoring via tank water. The application of this sensor was demonstrated in fish stress studies, involving acute stress challenges either by applying handling (chasing and air exposure) [12] or higher tank stocking densities [48] that trigger the release of cortisol into the bloodstream and surrounding water. HPLC was used to validate the aptasensor detection outcome, with good agreement between the two methods.

## 2. Materials and Methods

### 2.1. Materials

Absolute ethanol, arginine, ß-estradiol-6-(O-carboxy-methyl)oxime:BSA, calcium chloride dihydrate (CaCl_2_·2H_2_O), lysine, gold (III) chloride solution, 4-(2-hydroxyethyl)-1-piperazineethanesulfonic acid (HEPES), hydrochloric acid (HCl), hydrocortisone, magnesium chloride (MgCl_2_), magnesium chloride hexahydrate (MgCl_2_·6H_2_O), magnesium sulfate heptahydrate (MgSO_4_·7H_2_O), sodium bicarbonate (NaHCO_3_), sodium citrate tribasic dihydrate, sodium chloride (NaCl), sodium phosphate monobasic monohydrate, Tris(hydroxymethyl)aminomethane (Tris), phosphate-buffered saline (PBS), tris(2-carboxyethyl)phosphine (TCEP), sodium hydroxide (NaOH) and single-stranded oligonucleotides were purchased from Sigma-Aldrich. The sequence of anti-cortisol DNA aptamers is shown in Table 1. For conjugation to gold nanoparticles, the selected aptamer sequence was modified with a thiol group (thio C6 linker) at the 5′end (Sigma Aldrich, Burlington, MA, USA). CALNN peptide (>95%) was obtained from SABio.

### 2.2. Preparation of Artificial Seawater (ASW)

The composition of artificial seawater (35 ppt salinity) was prepared according to a reported protocol [49]. To 500 mL of water, 14.16 g NaCl, 2.74 g MgCl_2_·6H_2_O, 3.7 g MgSO_4_· 7H_2_O, 0.55 g CaCl_2_·2H_2_O, 0.39 g KCl and 0.10 g NaHCO_3_ were added. The resulting mixture was sonicated, filtered, and stored at 4 °C for further use.

### 2.3. AuNP Synthesis

Citrate coated AuNPs were synthesized by the reduction of HAuCl_4_, according to a reported protocol [50]. Experimentally, gold (III) chloride solution (1 mM, 50 mL) was brought to the boil before the quick addition of sodium citrate solution (40 mM, 5 mL). The reaction mixture was refluxed at 110 °C for 30 min and then cooled to room temperature. The resulting AuNPs were used for further conjugation and sensing studies.

### 2.4. Aptamer Affinity Characterization Using Bare AuNPs’ Aggregation Profile

A cortisol stock solution (1 mM) was prepared by dissolving solid cortisol in absolute ethanol. Lower cortisol concentrations were obtained by diluting the cortisol stock into cortisol binding buffer (50 mM Tris, 137 mM NaCl, 5 mM MgCl_2_ for Apt 15-1a, and 20 mM sodium phosphate monobasic monohydrate, pH 7.5, 2 mM MgCl_2_ for Apt CSS.1), or into PBS (0.01 M). For each dilution step, the cortisol solution was thoroughly mixed and spun down to prevent adhesion on the side walls of tubes.

The assay was performed by incubating Apt 15-1a or Apt CSS.1 (10 µM, 10 µL) with varying cortisol concentrations (0.1–10,000 nM, 10 µL) in respective cortisol binding buffers at room temperature for 15 min. The as-prepared AuNP (11.6 nM, 70 µL) was then added to the aptamer–cortisol complex. Finally, PBS (0.1 M, 10 µL) was mixed into the reaction mixtures and their absorbance spectra were acquired (Synergy 2; BioTek) after 15 min. The differential stability of AuNPs in the absence or presence of cortisol was measured by the absorbance intensity ratio of 520 nm/650 nm (A_520_/A_650_).

### 2.5. Co-Conjugation of Aptamer and Blocker on AuNPs (Au-Apt-CALNN) and Stability Test

AuNPs were co-conjugated to Apt thiol-CSS.1 and CALNN peptide using our developed protocol. To reduce the disulfide bonds in the aptamer, Apt thiol-CSS.1 (100 µM, 20 µL) was mixed into Tris (2-carboxyethyl) phosphine (5 mM, 4 µL) and HEPES buffer (5 mM, 76 µL) at 700 rpm for 10 min. The mixture was then transferred into a 3 kDa spin filter (PALL) and centrifuged at 12,500 rpm for 7 min, three times. The resulting supernatant was resuspended in DI water, yielding the thiol-active aptamer for conjugating onto AuNP.

To 908 µL of AuNP, 56 µL of the thiol-active Apt CSS.1 and 10 µL of HCl (0.1 M) were sequentially added. The Au-Apt solution was mixed at 500 rpm for 15 mins and left to incubate overnight. CALNN peptide (1.8 mM, 60 µL) was then added to the Au-Apt solution and mixed at 500 rpm for 15 min. Finally, NaOH (0.1 M, 10 µL) was added, and the reaction mixture was vortexed. The conjugate was centrifuged at 10,000 rpm for 45 min and resuspended in water for further use.

The stability of nanoparticles at each preparation stage was assessed by mixing Au-Apt or Au-Apt-CALNN (60 µL) into 21 µL of DI water and 9 µL of salt water, i.e., artificial seawater (ASW), Recirculating Aquaculture System (RAS) tank water, or PBS. Their absorbance spectra were acquired using a microwell plate reader, and quantitative evaluation was performed by calculating the absorbance ratios at 525 nm and 650 nm (A_525_/A_650_).

### 2.6. Cortisol Detection Using Au-Apt-CALNN

The Au-Apt-CALNN conjugate (60 µL) and cortisol of varying concentrations (0.1–1000 nM, 20 µL) in PBS were incubated at room temperature for 20 min. MgCl_2_ of varying concentrations (0.01 M, 0.05 M or 0.1 M, 10 µL) were then added to the above reaction mixture and the absorbance spectrums were acquired at different time intervals. The differential aggregation profile between Au-Apt-CALNN and cortisol-bound Au-Apt-CALNN were quantified by the ratio of the absorbance intensity at 525 nm relative to the absorbance intensity at 650 nm (A_525_/A_650_). For evaluating the selectivity of Au-Apt-CALNN, the cortisol analyte was replaced by either ß-estradiol-6-(O-carboxy-methyl) oxime:BSA, arginine or lysine. Fish tank water samples were filtered through a hydrophobic PTFE filter with pore sizes of 0.22 µM before 10 times dilution into PBS (0.01 M).

### 2.7. Dynamic Light Scattering Characterization

DLS tests were performed using a Malvern Zetasizer (Nano S90) to measure the sizes of Au, Au-Apt and Au-Apt-CALNN in water. The sizes of Au-Apt-CALNN and those incubated with cortisol were measured in PBS and with/without MgCl_2_ addition. The reported DLS sizes were an average of 3 acquisitions.

### 2.8. Fish Tank Water Samples

Water samples were collected from a Recirculating Aquaculture System (RAS) housing healthy Asian sea bass sourced from local commercial fish farms at the Aquaria of Republic Polytechnic, Singapore. Fish were subjected to acute stress challenges by handling or increasing stocking densities as approved by the Republic Polytechnic’s Institutional Animal Care and Use Committee (IACUC Protocol #2022/RP/00001). The handling (chasing by net and air exposure) stress challenge was conducted in a 3000 L tank integrated in a 9000 L RAS system circulating 30 ppt saltwater suitable for Asian sea bass, as described in Tan et al. [12], where fish were subjected to periods of chasing of progressively increasing durations (e.g., 5 min, then 10 min then 15 min) using two large nets, followed by air exposure (i.e., removal from water) for 10 s. Water samples were siphoned out from the tank at the end of the chasing periods (named “Chase 5 min” and “Chase 30 min” after the 5 min and 30 min (5 + 10 + 15 min) accumulated chasing periods, respectively), and at several time points (2 h, 4 h and 24 h) from the end of the last chasing period (named “Post-chase 2 h”, “Post-chase 4 h” and “Post-chase 24 h”). Fish were maintained at a tank stocking density of 2.094 kg/m^3^.

Separately, in a stocking density challenge, Asian sea bass were raised in two RAS systems, system 1 (S1) and system 2 (S2), with system volumes of 496 L and tank volumes of 178 L (two tanks in each system). The S1 system stocking density was 22.4 kg/m^3^ while the S2 system stocking density was higher at 37.2 kg/m^3^. Similarly, water samples were siphoned out from S1 and S2.

### 2.9. High Performance Liquid Chromatography (HPLC) for Analysis of Cortisol in Fish Tank Water

The cortisol measurements from all fish tank water samples were validated by high-performance liquid chromatography (HPLC) with prior liquid–liquid extraction according to reported methods [12,51,52]. Liquid–liquid extraction was performed by mixing equal volumes of dichloromethane and water samples for 10 min, followed by evaporation of the organic phase using a rotary evaporator (IKA RV 10, IKA-Weke Gmbh & Co. KG, Staufen, Germany). The resulting precipitate was then reconstituted in a 50% methanol solution, filtered through a 0.22 µm PVDF filter, and analysed using HPLC (Shimadzu LC-2050C 3D, Kyoto, Japan). The HPLC measurement was conducted through a Shim-Pack GIST C18 normal-phase column coupled to a photodiode array (PDA) detector set at 245 nm with an isocratic flow of methanol and 10 mM of ammonium formate in water as mobile phases. The HPLC was calibrated to cortisol standards (1 pM to 10 µM) in a 50% methanol solution. Internal standards (500 µL of 1 µM cortisol) were added into all water samples and cortisol calibration standards.

## 3. Results and Discussion

### 3.1. Confirmation of Aptamer–Cortisol Binding Using Bare AuNPs

As described in the Introduction, Apt 15-1 and one of its derivatives (15-1a) have been used for developing cortisol biosensors, exploiting different sensing principles, such as AuNP-based colorimetric [30,31,37,53] and electrochemical modes [2,54,55,56]. These studies were driven by pioneer reports that the above aptamers bind to cortisol with high selectivity and sensitivity [30,31,37]. However, the complex between Apt 15-1a and cortisol was also found to be unstable (with a higher binding energy) [39]. This finding has also been supported by another study demonstrating negligible binding between Apt 15-1a and cortisol under the respective binding conditions [38]. To address the discrepancy, we first studied Apt 15-1a binding to cortisol using bare AuNPs’ aggregation profile, in comparison with a high-affinity aptamer Apt CSS.1 reported from simulations [38,40,42]. This binding study is based on the notion that single-stranded DNA aptamers can protect citrate ion-coated AuNP from salt-induced aggregation; but such protection is reduced when the aptamer is complexed with cortisol in their reported optimized binding buffer compositions (50 mM Tris, 137 mM NaCl, 5 mM MgCl_2_ for Apt 15-1a and 20 mM sodium phosphate monobasic monohydrate, pH 7.5, 2 mM MgCl_2_ for Apt CSS.1) [31,37,38]. Such a detection principle is proven valid because cortisol itself does not have strong interactions with AuNP [42].

**Figure 1:**
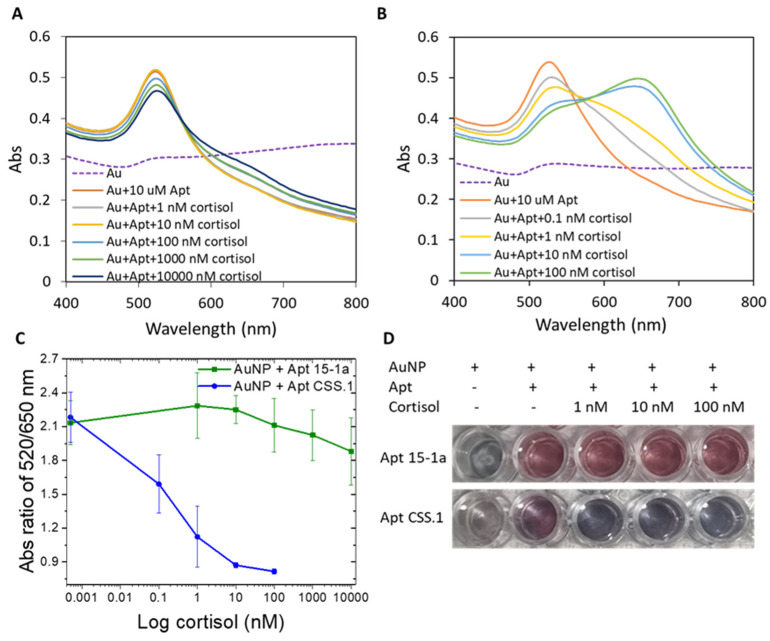
Absorbance spectrum of AuNP with (**A**) Apt 15-1a, before and after cortisol addition (1–10,000 nM) and (**B**) Apt CSS.1 before and after cortisol addition (0.1–100 nM). (**C**) The absorbance ratio of A_520_/A_650_ of the two cases (**A**,**B**) as a function of cortisol concentrations. (**D**) Photographic images of the AuNP solutions with the two aptamers, before and after cortisol addition (1–100 nM).

As shown in Figure 1, when cortisol in concentration ranges of 10–10,000 nM and 0.1–100 nM, respectively, was added to Apt 15-1a and CSS.1 protected AuNPs in a 1:1 mixture of cortisol binding buffer and PBS, AuNPs became more and more aggregated in both cases (Figure 1A,B). This cortisol concentration-dependent AuNP aggregation confirmed the formation of the aptamer–cortisol complex for both Apt 15-1a and CSS.1. The plots of the A_520_/A_650_ ratio (the degree of aggregation) as a function of cortisol concentration (Figure 1C) show clearly that the degree of AuNP aggregation (or the degree of complex formation) is much larger for CSS.1 than 15-1a. Visually, the color change with the addition of 1–100 nM of cortisol is also more prominent for Apt CSS.1 than Apt 15-1a (Figure 1D). With this experiment, we confirmed a higher cortisol binding affinity of the Apt CSS.1 aptamer than the Apt 15-1a under the binding buffer used in the current study. This observation concurs with earlier reports that Apt 15-1a has negligible cortisol binding relative to Apt CSS.1 [38,42].

### 3.2. Co-Conjugation of Aptamer and Blocker Molecules with AuNPs and Particle Stability Test

With the confirmation of the higher affinity of 42 mer Apt CSS.1 than 15-1a to cortisol, we proceeded to conjugate the CSS.1 aptamer onto AuNPs (Au-Apt) via thiol-mediated bonds. The UV-vis spectrum shows ~3 nm red shift for Apt CSS.1-conjugated AuNPs (Figure S1A), a primary indication of the aptamer attachment to the particles. From DLS measurements (Table 2), the hydrodynamic size of AuNPs after this aptamer conjugation increased from 15.2 ± 0.1 nm to 22.5 ± 0.1 nm, further confirming the successful aptamer conjugation. The stability of the as-prepared Au-Apt against salt-induced aggregation was assessed in various mediums, such as 1× PBS, Recirculating Aquaculture System (RAS) tank water of 30 ppt salinity, and artificial seawater (ASW) of 35 ppt salinity by UV-Vis spectroscopy (Figure S1). In these media, the UV-vis spectrum of the Au-Apt conjugate obviously red-shifted, with the appearance of peaks at longer wavelengths (Figure S1B–D) relative to that in water, indicative of particle aggregation to certain degree. The associated absorbance ratio (A_520_/A_650_), a measure of the degree of aggregation, decreased from 7.9 (in water) to 4.4, 3.3 and 2.7, respectively. The more and more drastic aggregation is attributed to the increase in ionic strengths of the salt mediums.

The depleted stability of the Au-Apt in salt mediums provided the impetus for us to co-conjugate a blocker molecule (CALNN) to improve the stability of the nanoparticles. The amphiphilic nature of CALNN from its hydrophobic alanine and leucine chains, with hydrophilic asparagine and carboxylic acid terminal groups also promotes high biocompatibility in both aqueous and organic mediums [57,58,59]. The CALNN peptide was conjugated onto Au-Apt through thiol-Au interaction, where the thiol group is from the cysteine (Figure S2). The UV-vis spectrum of the Au-Apt-CALNN shows a further peak absorbance shift from 523 nm (AuNP-Apt) to 525 nm (Au-Apt-CALNN), which is primary evidence for the attachment of the CALNN molecules (Figure S1A). The associated A_525_/A_650_ ratio in water is 6.5. The hydrodynamic size increased from 22.5 ± 0.1 nm for Au-Apt to 27.8 ± 0.7 nm for Au-Apt-CALNN (Table 2), which further supports the attachment of CALNN. The Au-Apt-CALNN exhibited much higher stability than Au-Apt against salt-induced aggregation in PBS, RAS tank water, and ASW (Figure S1B–D), with A_525_/A_650_ ratios of 6.3, 4.0 and 3.8, respectively, for Au-Apt-CALNN.

Among the three salt mediums tested, the ASW formulation used for marine culture [49] was found to have the highest ionic strength and salinity (35 ppt), even higher than the real sample matrix of the RAS tank water (30 ppt). The UV-Vis characterization shows that the CALNN-stabilized Au-Apt-CALNN conjugate retained its dispersity in PBS to almost the same extent as in water (A_525_/A_650_ ratios of 6.5 and 6.3). Furthermore, the 10%-diluted RAS tank water or ASW in PBS also did not disturb Au-Apt-CALNN particles’ stability (Figure S3). This is an important characteristic of the particles to be used for cortisol detection in PBS buffer.

### 3.3. Cortisol Detection Using Au-Apt-CALNN Conjugate

**Figure 2:**
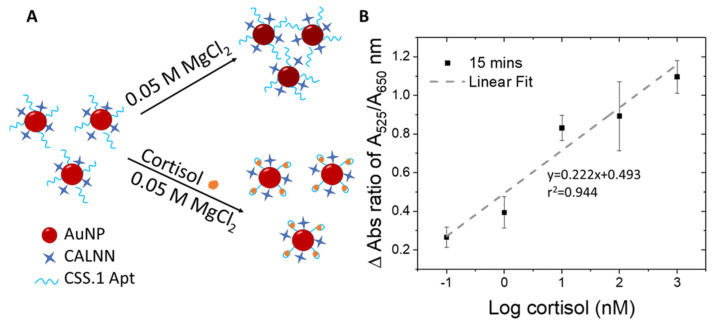
(**A**) Schematic illustration of the detection principle using Au-Apt-CALNN. (**B**) Plot of the change in the absorbance ratio of A_525_/A_650_ as a function of log cortisol (nM).

The Au-Apt-CALNN was used to detect cortisol, by exploiting the difference in the colloidal stability of the nanoparticles in a stronger salt condition (MgCl_2_ added into the reaction solution), where the attached aptamer may adopt a different folding configuration upon cortisol binding (Figure 2A). In comparison to monovalent cations, multivalent metal ions such as Mg^2+^ have more positive charges and can induce stronger charge screening effects and absorbance changes in AuNPs [60]. We optimized the concentration of MgCl_2_ and the incubation time that allowed the largest differentiation in the A_525_/A_650_ ratio within a reasonable reaction time suitable for on-site detection.

Experimentally, MgCl_2_ of 0.01, 0.05 or 0.1 M were added to the reaction solution containing 0, 1, and 100 nM cortisol, followed by UV-vis spectrum-acquisition at time intervals of 5, 10, 15, and 20 min to determine the optimal MgCl_2_ concentration (Figures S4–S6). At the lowest MgCl_2_ concentration of 0.01 M (Figure S4), changes in absorbance spectrum were only detectable for 100 nM cortisol as compared to the control sample (0 nM of cortisol) over the tested time frames from 5 to 20 min. On the other hand, when the MgCl_2_ concentration was increased to 0.1 M (Figure S6), changes in A_525_/A_650_ ratio can be observed for 1 nM cortisol from 15 min onward (Figure S6). The differential stability of Au-Apt-CALNN particles with versus without cortisol increases at higher salt concentrations (0.1 M versus 0.01 M MgCl_2_), especially with longer reaction times from 15 to 20 min (Figures S4 and S6). However, at 0.1 M MgCl_2,_ there was no differentiable response between 1 nM and 100 nM cortisol. The reduced resolution could be attributed to the kinetically fast reaction promoted by high MgCl_2_ concentration.

Notably, at MgCl_2_ concentrations of 0.05 M, 1 nM, and 100 nM, cortisol led to differential responses in UV-vis spectra and A_525_/A_650_ ratios at 10 and 15 min (Figure S5) relative to the control (no cortisol). We thus defined 0.05 M MgCl_2_ as the optimal concentration and 15 min as the optimal reaction time. Under these conditions, UV-vis spectra for 0–1000 nM cortisol were acquired (Figure S7). Relative to the control spectrum (dotted line for no cortisol), all spectra with cortisol exhibited higher peak absorbances, indicative of higher stability in a cortisol concentration-dependent manner.

To eliminate potential interference by the water matrix, the change in absorbance ratio (delta A_525_/A_650_) was calculated by subtracting the A_525_/A_650_ of the control sample (no cortisol) from those with cortisol of various concentrations. The delta A_525_/A_650_ ratios were plotted as a function of logarithmic cortisol concentrations (Figure 2B), yielding an r^2^ value of 0.944. The r^2^ value of our assay (0.944) is comparable to the reported colorimetric detection of hormones using AuNP-aptamer based assays (r^2^ = 0.9209 – 0.94) [61,62]. The LOD obtained for the sensing assay is 100 pM and can be completed in as fast as 15 minutes. Compared to the pioneer report of using this absorbance ratio of DNA aptamer-AuNP conjugate for quantification of adenosine (0.5 units) [46], the absolute A_525_/A_650_ change measured in our study for cortisol was significantly larger (0.8 units), which assures us of the reliability of this sensing method.

To understand the interaction (i.e., configuration of aptamer before and after analyte binding) and colloidal stability of Au-Apt-CALNN and cortisol (Au-Apt-CALNN/cortisol), DLS measurements were further performed under the salt conditions used for cortisol detection (before and after addition of MgCl_2_) (Table 2). Before MgCl_2_ addition, the size of Au-Apt-CALNN was reduced from 27.8 ± 0.7 nm (in water) to 22.9 ± 0.5 nm (in PBS). This could be because in water the negative charge distribution along the aptamer backbone creates electrostatic repulsive forces between individual chains, which facilitates chain extension, while in a salt medium, such as PBS, the electric double layer of the aptamer chains is suppressed, which reduces electrostatic repulsive forces [46]. Hence, the aptamer collapses back to the surface of AuNP, resulting in smaller particle sizes and a thinner aptamer layer [46]. After cortisol binding in PBS, the size of Au-Apt-CALNN/cortisol (25.4 ± 0.4 nm) is not significantly different from that before being cortisol bound (22.9 ± 0.5 nm). After the addition of MgCl_2_, the size of Au-Apt-CALNN greatly increases to 49.2 ± 13.8 nm from that in PBS (22.9 ± 0.5 nm). Such a drastic increase in size and size distribution indicates the occurrence of aggregation of the nanoparticles due to stronger aggregation force from the bivalent salt. Under MgCl_2_, when cortisol binds to the aptamer, the size of the Au-Apt-CALNN/cortisol (40.2 ± 1.9 nm) is decreased relative to Au-Apt-CALNN without cortisol binding (49.2 ± 13.8 nm). This is an indication that the aptamer strains become folded and less extended from the surface of the nanoparticles. The more compact Apt–cortisol complex may exert a more rigid conformation, leading to more enhanced steric stabilization than that of an unfolded flexible aptamer without cortisol. This observation is in line with the earlier report on the adenosine AuNP sensor using an anti-adenosine aptamer [46].

### 3.4. Selectivity Test and Real Sample Test

**Figure 3:**
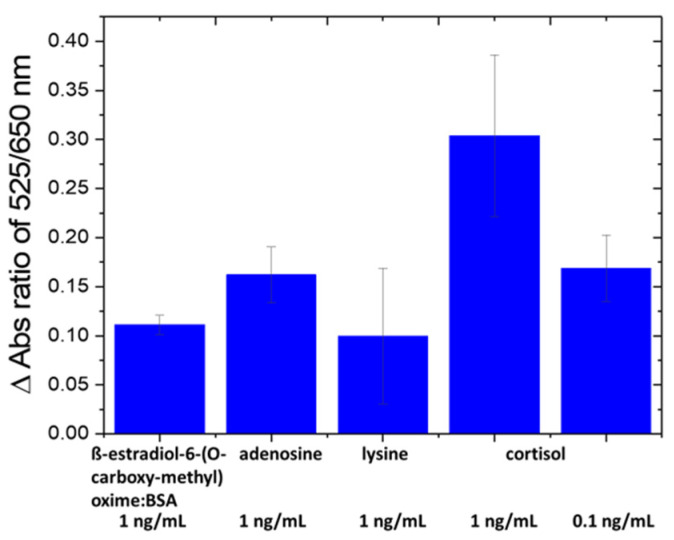
Selectivity test of Au-Apt-CALNN against other interfering molecules.

The selectivity of the nanosensor was evaluated against amino acids [43] and proteins in fish tank water, such as ß-estradiol-6-(O-carboxy-methyl) oxime/BSA, arginine and lysine at 1 ng/mL (Figure 3). The specificity of the nanosensor was confirmed since the change in absorbance ratio of A_525_/A_650_ caused by interfering species is lower than that by cortisol at a 10× lower concentration (0.1 ng/mL). The ng/mL units were used for all compounds and/or their BSA conjugate for comparison.

**Figure 4:**
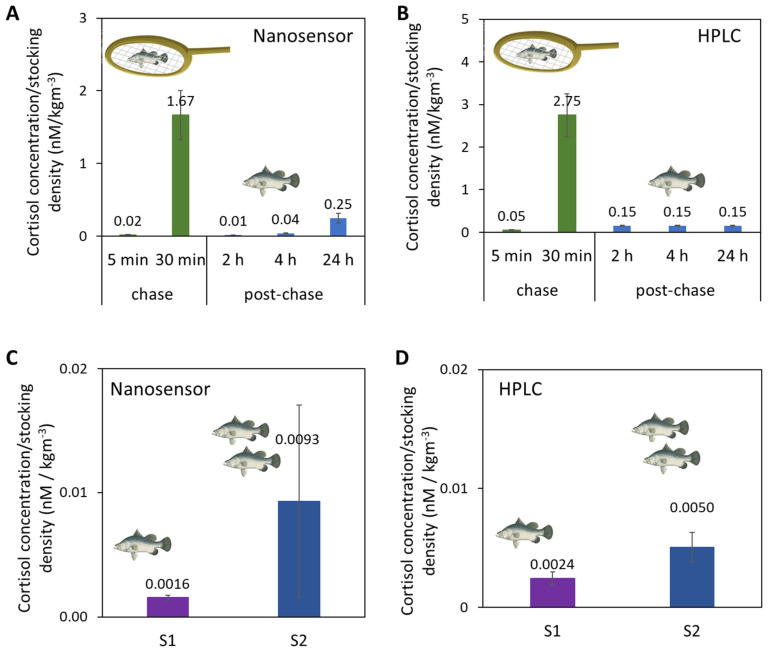
Cortisol concentrations normalized to the system stocking density in fish tank water samples from a handling stress experiment by (**A**) Au-Apt-CALNN nanosensor and (**B**) HPLC, and from a stocking density experiment by (**C**) Au-Apt-CALNN nanosensor and (**D**) HPLC.

Upon confirmation of its selectivity, we then used the Au-Apt-CALNN nanosensor to detect cortisol from fish tank water samples where the fish were challenged by either handling or increased stocking density. The results were validated with HPLC. First, in the handling stress (chasing and air exposure) experiments, the fish tank water samples were collected at the ends of chasing periods (5 min and 30 min accumulated chasing) and at different time points post-chasing (2 h, 4 h and 24 h) for cortisol quantification by the nanosensor (Figure 4A) and HPLC (Figure 4B). As the number of fish in the tank affected the concentration of cortisol released in the water, the cortisol concentrations in this comparison (and the 2nd stress study below) were normalized to the stocking densities of fish in the tank (2.094 kg/m^3^). Both the nanosensor and HPLC confirmed the drastic increase of cortisol levels after 30 min of chasing, and the subsequent decrease 2 h post-chase.

In a second stress study, the stocking densities of fish tank waters were varied, as high stocking density is known to induce chronic stress in fishes and negatively affects fish growth and feed utilization. [48,63,64] In this study, water samples were collected from fish raised in two RAS systems of different system stocking densities. System 1 (S1) had a lower system stocking density (22.4 kg/m^3^) than System 2 (S2) (system: 37.2 kg/m^3^). As measured by nanosensor and HPLC, the cortisol concentrations normalized to the system stocking density in S1 were lower than for S2 (Figure 4C,D). Quantitative comparison of all the fish tank water samples from the handling and stocking density experiments shows a correlation of r^2^ = 0.9851 between the nanosensor versus HPLC (Figure S8). The consistent results of our nanosensor relative to HPLC suggest the potential of this aptasensor to be used as a detection tool to study fish stress on-site in farms. Based on the aptasensor LOD and stability in complex matrices (like fish tank water), this aptasensor also has the potential for cortisol detection in human samples or other animal samples, which generally have higher cortisol concentrations than fish tank water [8].

## 4. Conclusions

We have developed and validated an aptasensor for non-invasive detection of fish stress via cortisol measurements from fish tank water. We first identified a high affinity aptamer and developed a conjugation method involving a short peptide blocker molecule to gain high stability for the particle when exposed to fish tank or artificial seawater. We then developed a sensing protocol involving an optimal MgCl_2_ concentration that can drive more aggressive aggregation in the nanoparticle–aptamer conjugate before cortisol binding, but not after cortisol binding. The differential aggregation profile is quantitatively correlated to the cortisol concentration. The sensing mechanism related to the folding of the aptamer upon cortisol binding, and the associated increased protection from MgCl_2_-induced aggregation was deeply characterized with particle size analysis by UV-vis and DLS analysis. The nanosensor exhibited an LOD of 100 pM with a 35-minute turnover time (20 min incubation between Au-Apt-CALNN and cortisol, followed by 15 min incubation after adding MgCl_2_), which is suitable for fast on-site cortisol detection from fish tank water. The nanosensor was validated using HPLC with well-correlated results. The aptasensor will be useful for non-invasive, frequent, and rapid fish stress monitoring. This sensor may also be potentially useful for stress quantification in many other species or biological contexts.

## Figures and Tables

**Table 1:** Sequence of single-stranded oligonucleotides purchased from Sigma-Aldrich.

Aptamer (Apt)	Sequence 5′-3′
15-1a	ATGGGCAATGCGGGGTGGAGAATGGTTGCCGCACTTCGGC
CSS.1	GACGACGCCCGCATGTTCCATGGATAGTCTTGACTAGTCGTC

**Table 2:** DLS measurements of nanoparticles sizes.

	In Water (nm)	In PBS (nm)	In PBS and MgCl_2_ (nm)
Au	15.2 ± 0.1	-	-
Au-Apt	22.5 ± 0.1	-	-
Au-Apt-CALNN	27.8 ± 0.7	22.9 ± 0.5	49.2 ± 13.8
Au-Apt-CALNN + cortisol *	-	25.4 ± 0.4	40.2 ± 1.9

* The concentration of cortisol used is 100 nM.

## Data Availability

The original contributions presented in the study are included in the article/Supplementary Material, further inquiries can be directed to the corresponding author/s.

## Supplementary Materials

The following supporting information can be downloaded at: https://www.mdpi.com/article/10.3390/biom14070818/s1, Figure S1: (**A**) Normalized UV-vis spectrums of AuNP in water (red), Au-Apt (blue) and Au-Apt-CALNN (orange). (**B**–**D**) Absorbance spectrums of Au-Apt (blue) and Au-Apt-CALNN (orange) in (**B**) PBS buffer, (**C**) Recirculating Aquaculture System (RAS) tank water and (**D**) artificial seawater (ASW); Figure S2: Conjugation mechanism of CSS.1 Apt and CALNN peptide on AuNP; Figure S3: Normalized UV-vis spectrums of Au-Apt-CALNN in 1× PBS (blue), 10% RAS tank water in 1xPBS (orange) and 10% ASW in 1× PBS (grey); Figure S4: UV-Vis spectrums acquired at different time intervals of (**A**) 5 min (**B**) 10 min (**C**) 15 min (**D**) 20 min, after adding 0.01 M MgCl_2_; Figure S5: UV-Vis spectrums acquired at different time intervals of (**A**) 5 min (**B**) 10 min (**C**) 15 min (**D**) 20 min, after adding 0.05 M MgCl_2_; Figure S6: UV-Vis spectrums acquired at different time intervals of (**A**) 5 min (**B**) 10 min (**C**) 15 min (**D**) 20 min, after adding 0.1 M MgCl_2_; Figure S7: UV-vis spectra of Au-Apt-CALNN in the presence of 0 to 1000 nM cortisol; Figure S8: Correlation between nanosensor versus HPLC for all fish tank water samples data.
