# Supplementary material for: A Gold Nanoparticle-Based Cortisol Aptasensor for Non-Invasive Detection of Fish Stress
Source: Biomolecules. 2024 Jul 9;14(7):818. doi: 10.3390/biom14070818 (PMC11274556; doi:10.3390/biom14070818)
Supplement: Supplementary file 1 [file biomolecules-14-00818-s001.zip › biomolecules-3037521-supplementary.pdf]

Supporting Information

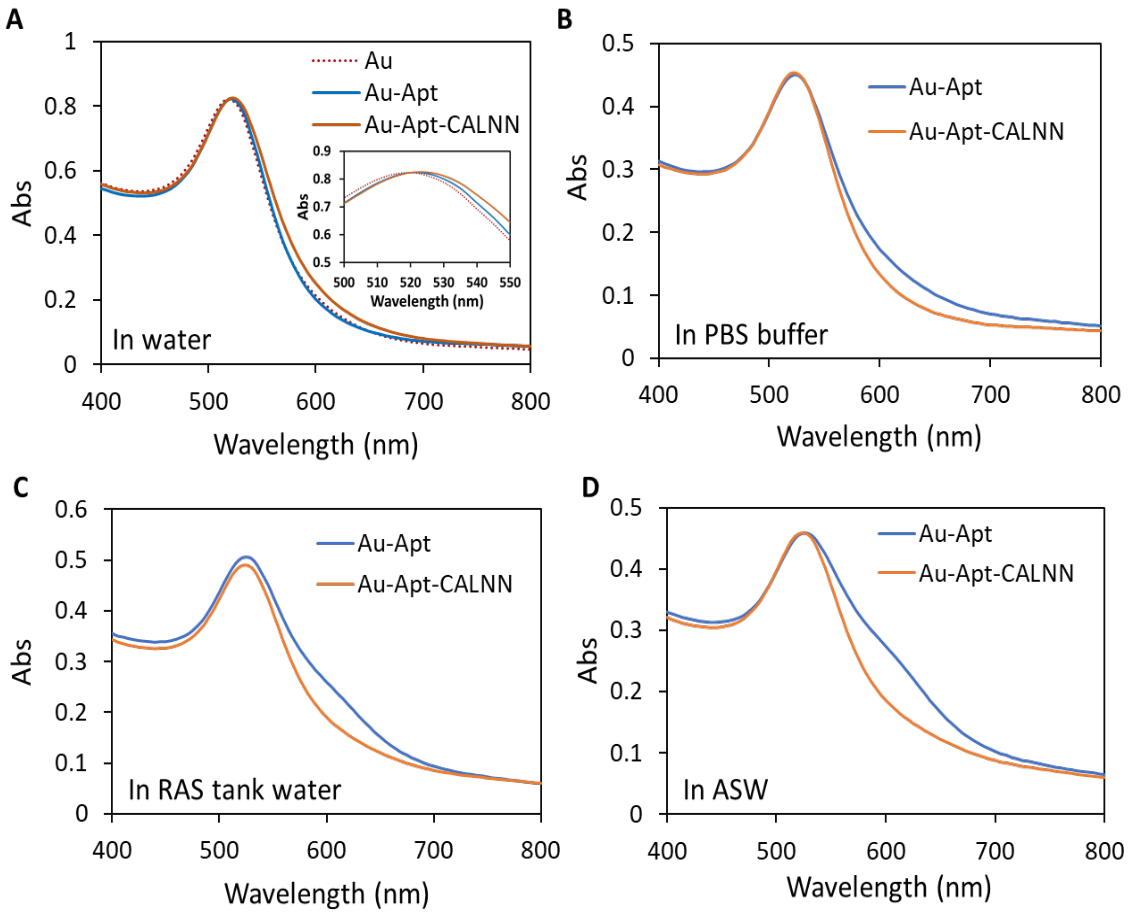

Figure S1. (A) Normalized UV-vis spectrums of AuNP in water (red), Au-Apt (blue) and Au-Apt-CALNN (orange). (B-D) Absorbance spectrums of Au-Apt (blue) and Au-Apt-CALNN (orange) in (B) PBS buffer, (C) Recirculating Aquaculture System (RAS) tank water and (D) artificial seawater (ASW).

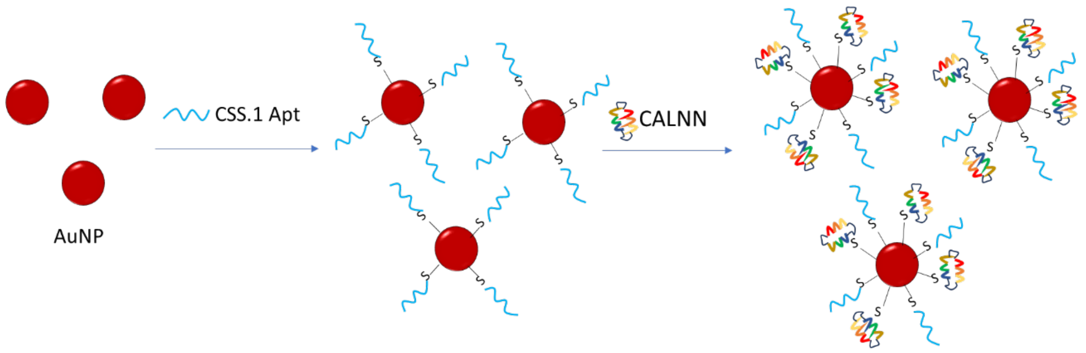

Figure S2. Conjugation mechanism of CSS.1 Apt and CALNN peptide on AuNP

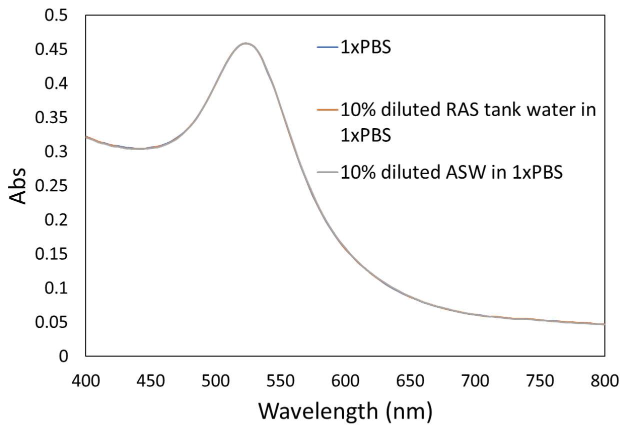

Figure S3. Normalized UV-vis spectrums of Au-Apt-CALNN in 1xPBS (blue), 10% RAS tank water in 1xPBS (orange) and 10% ASW in 1xPBS (grey).

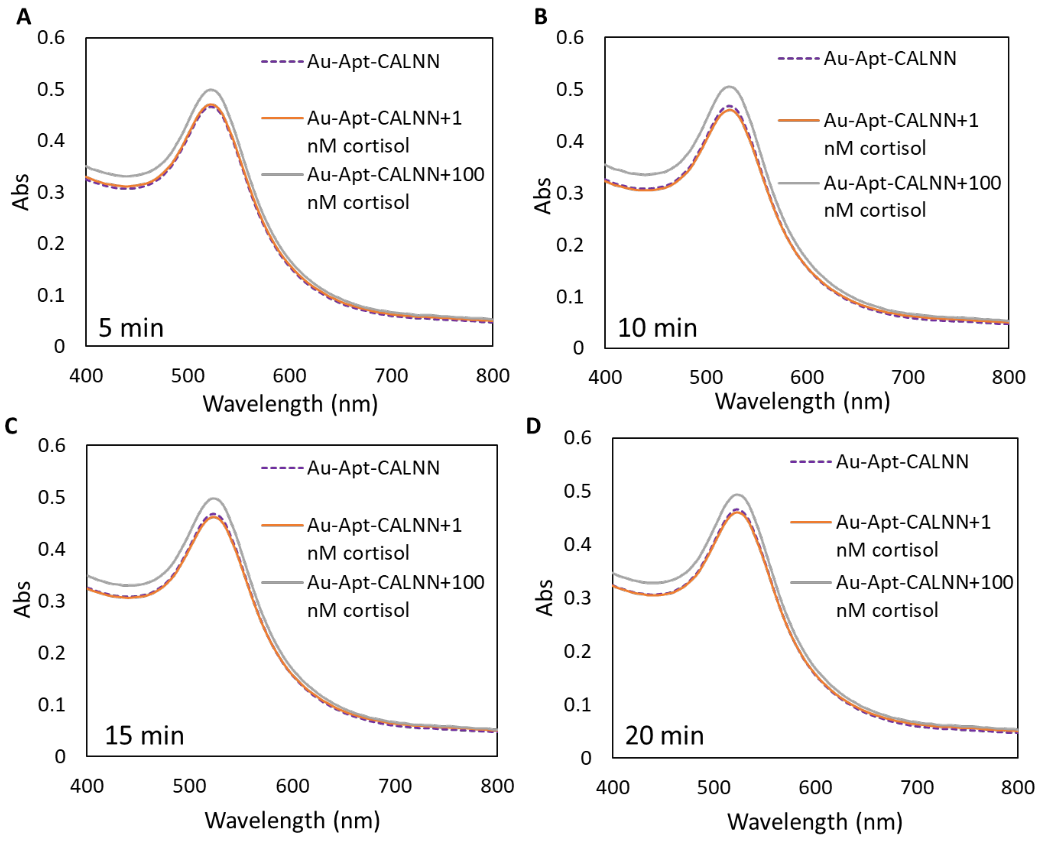

Figure S4. UV-Vis spectrums acquired at different time intervals of A) 5 min B) 10 min C) 15 min D) 20 min, after adding 0.01 M $MgCl_2$

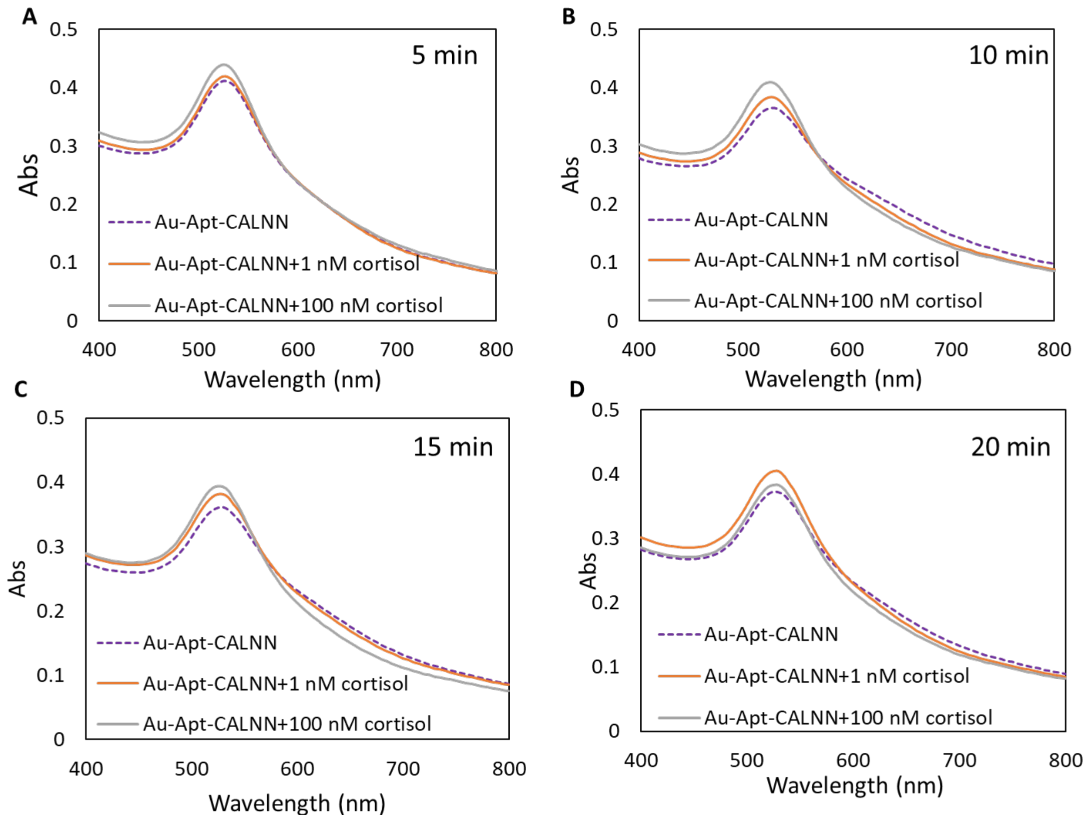

Figure S5. UV-Vis spectrums acquired at different time intervals of A) 5 min B) 10 min C) 15 min D) 20 min, after adding 0.05 M $MgCl_2$

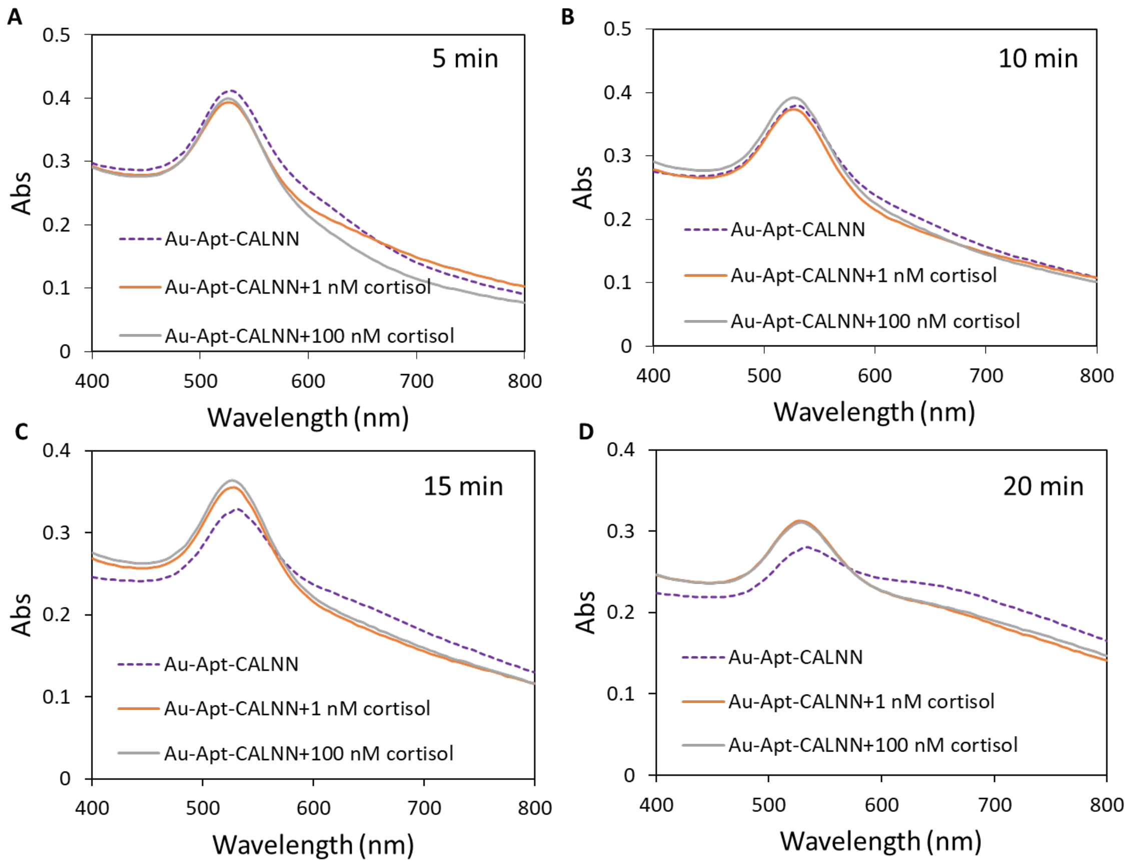

Figure S6. UV-Vis spectrums acquired at different time intervals of A) 5 min B) 10 min C) 15 min D) 20 min, after adding 0.1 M $MgCl_2$.

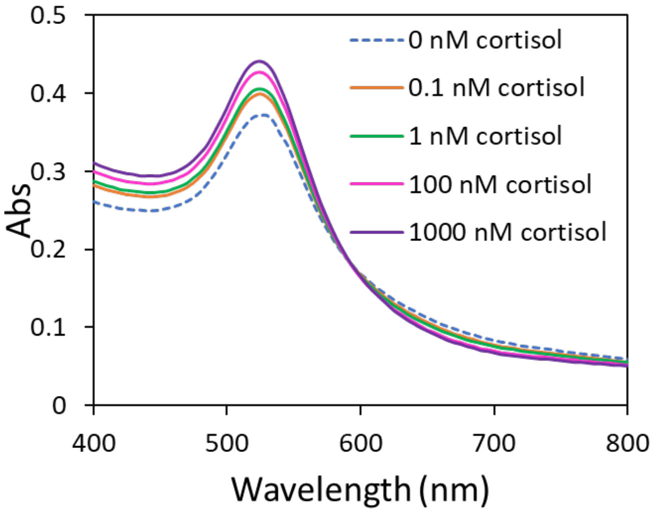

Figure S7. UV-vis spectra of Au-Apt-CALNN in the presence of 0 to 1000 nM cortisol.

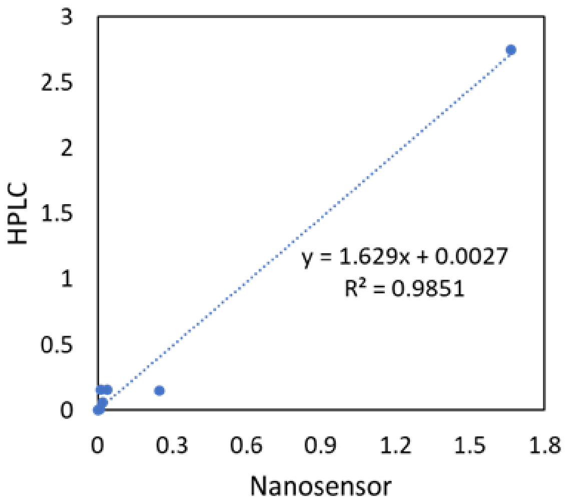

Figure S8. Correlation between nanosensor versus HPLC for all fish tank water samples data.
